# Correlation Analyses of Amylase and Protease Activities and Physicochemical Properties of Wheat Bran During Solid-State Fermentation

**DOI:** 10.3390/foods13243998

**Published:** 2024-12-11

**Authors:** Hongrui Ren, Tianli Wang, Rui Liu

**Affiliations:** State Key Laboratory of Food Nutrition and Safety, Tianjin University of Science and Technology, Tianjin 300457, China; rhr1122@163.com (H.R.); 13652104287@163.com (T.W.)

**Keywords:** wheat bran, solid-state fermentation, *Rhizopus oryzae*, amylase, protease

## Abstract

Solid-state fermentation (SSF) has emerged as an effective method for wheat bran valorization, providing advantages like cost reduction, decreased water usage, and enhanced product quality. In this study, wheat bran was fermented using *Rhizopus oryzae* to evaluate the extraction yield of soluble dietary fiber, the activities of protease and amylase, and the physicochemical characteristics of wheat bran during SSF. The findings demonstrated that the maximum yield of soluble dietary fiber was achieved after 120 h of fermentation at a moisture content of 55%. Simultaneously, protease activity peaked at 45% moisture content after 120 h, while amylase activity was maximized at 55% moisture content after 96 h. The microstructure result indicated that most of the starch granules degraded after 144 h of fermentation at a moisture content of 55%, exhibiting a smooth outer layer of wheat bran. Furthermore, fermented bran showed a significant rise in total phenols, peaking at 96 h at a moisture content of 55%. Flavonoid content also reached its maximum after 72 h of fermentation at 55% moisture content. The content of alkylresorcinols in fermented wheat bran changed slightly under different moisture content and fermentation time conditions, which was consistent with the change in pH value. The DPPH radical scavenging rate was optimal when the moisture content was 55% after 96 h. The ABTS radical scavenging rate, hydroxyl radical scavenging rate, and reducing ability were optimal at 55% moisture content after 120 h. These findings demonstrate that the optimal conditions for the SSF of wheat bran using *Rhizopus oryzae* involve maintaining the moisture at 55%, suggesting that this method is effective for enhancing the value of wheat bran.

## 1. Introduction

In the 2023–2024 period, wheat remained a crucial global food crop with an estimated production of 787.3 million tons worldwide [1]. During the process of milling wheat into flour, wheat bran emerges as the primary by-product, yielding about 32 million tons annually [2]. It is abundant in dietary fiber (DF), often accounting for more than 50% of its total weight [3]. Typically, DF is categorized into soluble (SDF) and insoluble (IDF) dietary fibers. In wheat bran DF, SDF and IDF make up about 5% and 95%, respectively [4]. Dietary fiber offers numerous health benefits, such as lowering the chances of heart disease, stroke, hypertension, diabetes, and obesity [5]. Specifically, SDF is crucial in enhancing the growth of intestinal probiotics; regulating blood sugar levels; decreasing lipid profiles, total serum cholesterol, and LDL cholesterol; and modulating the immune system [6,7]. By contrast, IDF primarily aids in improving bowel movements by easing the transit of materials through the gastrointestinal tract [8]. Since the existing level of wheat bran SDF including soluble arabinoxylan (AXs) and *β*-glucan is low, many modification methods have been utilized in order to improve the SDF release in wheat bran. Current approaches to bran modification comprise physical techniques (baking, steaming, steam explosion, extrusion), chemical treatments (alkali, acid, carboxymethylation, cross-linking), and biological methods that employ specific enzymes or microorganisms for enzymatic hydrolysis or fermentation of raw materials [9,10].

Biomodification is attracting significant interest because of its gentle processing conditions, efficiency, and eco-friendly nature. Notably, one application—solid-state fermentation (SSF)—has emerged as an effective method for generating value-added products, providing advantages like cost reduction, decreased water usage, and enhanced product quality [11]. *Rhizopus oryzae* fermentation increased wheat bran’s SDF content from 0.83% to 5.69%, improved pleasant flavor components such as 2-methybutyraldehyde, 2,3-pentanedione, *n*-hexanol, and newly produced vinyl acetate, and enhanced the extraction of antioxidant phenolic compounds [12,13,14]. In addition, for example, fermenting Proso millet bran with lactic acid bacteria via SSF enhanced the extraction yield of soluble dietary fiber from 4.2% to 7.6% [15]. Likewise, when wheat bran was fermented using lactic acid bacteria, the yield of soluble dietary fiber rose from 4.43% to 8.36%; fermentation with yeast increased it to 7.48% [16]. Furthermore, *Eurotium cristatum* fermentation increased wheat bran’s SDF content from 3.07% to 7.27% in just six days [17].

SSF is a complex heterogeneous reaction where water plays a crucial role. The enzymes produced by microorganisms during the SSF of wheat bran significantly affect its structure, bioactivity, and bioavailability [18]. Enzymatic activities vary with changes in moisture content and fermentation duration. For instance, fermenting wheat bran with *Bacillus* sp. TMF-2 at 50% moisture content resulted in peak α-amylase activity of 107 IU/g and mannanase activity of 7.2 IU/g, both observed on day 7. Cellulase activity reached a maximum of 4.7 IU/g on day 11, while the highest pectinase activity was 3.3 IU/g after fermenting for 10 days [4]. Another instance with Penicillium sp. FSDE15 at 60% moisture content revealed the highest endoglucanase yield of 17.92 ± 0.89 U/g at 168 h, total cellulase of 0.70 ± 0.12 U/g at 192 h, and β-glucosidase of 8.72 ± 0.42 U/g at 216 h of fermentation [19]. Conversely, fermenting wheat bran with *Aspergillus* at 70% moisture led to maximum feruloyl esterase activity of 1730 mU/g after 24 h; xylanase activity reached 868.1 U/g at 72 h, amylase activity peaked at 40.3 U/g following 96 h, and protease activity was 5.1 U/g at 72 h [20]. Specially, Aikat and Bhattacharyya investigated the extraction times of protease in SSF of wheat bran by *Rhizopus oryzae*, and the results indicated that repeated extractions performed three times was sufficient to extract nearly 90% of protease activity [21,22]. Ferreira et al. applied SSF to three agro-industrial by-products (brewers’ rice, corn grits, and wheat bran) by *Rhizopus oryzae*, aiming to enhance *α*-amylase activity [23]. Despite these results, how enzymatic activities vary with different moisture content and fermentation times during *Rhizopus oryzae* fermentation remains unclear.

In this study, we conducted a cumulative enzyme activity determination method to quantify the total enzyme activity, including free and absorbent enzyme activities, during solid-state fermentation of wheat bran by *Rhizopus oryzae*. The SDF yield, physicochemical properties (water activity, central temperature, moisture composition, total phenolic content, flavonoid content, alkylresorcinols content, and pH value), antioxidant activities (DPPH, ABTS, hydroxyl radical scavenging rates, and reducing ability), and microstructure were analyzed throughout the fermentation process. Moreover, the correlation analysis among the above various indicators was conducted with different moisture content and fermentation times. The methodologies in this study and its prospective results could lay theoretical support for understanding the mechanisms behind regulating high-solid fermentation efficiency and the application of high-quality dietary fiber from wheat bran.

## 2. Materials and Methods

### 2.1. Materials and Chemicals

Wheat bran (protein 14.64%, starch 16.29%, dietary fiber 48.46%) was provided by Fada Flour Co., Ltd. (Fada Flour Co., Ltd., Dezhou, China). *Rhizopus oryzae* was obtained from the Food Science and Engineering Laboratory at Tianjin University of Science and Technology, China, and stored in potato dextrose agar (PDA) plates. Unless otherwise stated, all chemicals and reagents used in this study were of analytical grade and were purchased from Sinopharm Chemical Reagent Co., Ltd. Company (Tianjin, China).

### 2.2. Sample Preparation

The preparation of fermented wheat bran followed the method described in a prior study [24]. After grinding, the dried wheat bran was passed through a 50-mesh sieve and placed into triangular flasks. Distilled water was added to adjust the substrate moisture to 35%, 45%, 55%, 65%, and 75%. Then, the samples were transferred into sterile containers and autoclaved at 121 °C for 20 min using a DSX-24L portable sterilizer (Shenan, Shanghai, China). Pre-cultured *Rhizopus oryzae* spores (10^6^–10^8^ cfu/mL) were added to the wheat bran at a 10% inoculum rate and incubated at 29 °C for 144 h. The fermentation was conducted under static conditions without extra mixing and aeration. During the fermentation process, samples of wheat bran were collected daily to measure the various indicators described below. Noticeably, the changes in central temperature and water activity during the fermentation process are described in the Supplementary Material.

### 2.3. Determination of SDF Yield

SDF from both unfermented and fermented wheat bran was extracted using a slightly modified version of the water extraction and alcohol precipitation method described in [25]. In summary, the samples were combined with distilled water at a 1:20 ratio, maintained at 85 °C with constant stirring for 2 h, and then centrifuged at 3500 rpm for 10 min. The supernatant was mixed with 95% (*v*/*v*) ethanol solution at four times its volume and left to precipitate overnight at 4 °C. After spinning at 3500 rpm for 20 min, the precipitate was dissolved again in distilled water and then freeze-dried to obtain SDF. The SDF extraction rate was measured as the proportion of SDF mass relative to the original wheat bran.

### 2.4. Enzyme Activity Detection

The crude enzyme extract was obtained following the procedure by Chen et al. with slight adjustments [26]. Initially, 50 mL of citrate buffer (50 mM, pH 5.0) was mixed with 5 g of wheat bran in a conical flask. The mixture was agitated in a thermostatic water bath shaker at 30 °C for 1 h at 180 rpm. Then, it was centrifuged at 4 °C and 4000 rpm for 20 min, after which the supernatant was collected. The precipitates were subjected to multiple extraction and centrifugation steps to obtain a collection of crude enzyme solution.

Amylase activity was determined by quantifying the reducing sugars released from the soluble substrate under conditions of pH 5.0 and 37 °C. One unit of amylase activity represents the enzyme amount needed to hydrolyze the substrate and release 1 mmol of glucose per minute at these conditions [27]. Protease activity was evaluated based on prior methods, adjusting the reaction temperature to 40 °C. In brief, 1 mL of casein solution (1%, *w*/*v*) and 1 mL of crude enzyme extract were incubated at 40 °C for 10 min. The reaction was stopped by the addition of 2 mL trichloroacetic acid (0.4 M), followed by centrifugation at 1000 rpm for 10 min at 4 °C. Next, 5 mL sodium carbonate (0.4 M) and 1 mL Folin and Ciocalteu’s reagent were added to 1 mL supernatant, and the reaction mixture was incubated for 20 min at 40 °C. The absorbance was recorded at 680 nm. One unit of protease activity was defined as the amount of enzymes required to liberate 1 μg of tyrosine under these assay conditions [28]. The protease activity of the sample was then calculated as follows:

(1) $$E\left(U/g\right)=\frac{A_{1}\times 4\times n}{m}\times \frac{1}{10}$$

where *E* is the activity of protease (U/g), *A*_1_ is the activity of enzyme in the final diluent of the sample obtained from the standard curve of tyrosine (U/mL), the total volume of reaction reagents was 4 mL, *n* is the dilution multiple of the sample, *m* is the mass of the sample (g), and reaction time was 10 min.

### 2.5. Microstructure

The wheat bran samples were uniformly attached to conductive tape and coated with a platinum layer under vacuum using the JEC-3000FC Auto Fine Coater (JEOL, Tokyo, Japan). The surface structure of the wheat bran was examined using a JSM-IT300LV SEM (JEOL, Tokyo, Japan) at 10 kV acceleration voltage and 1000× magnification.

### 2.6. Determination of Physicochemical Properties

#### 2.6.1. Total Phenolic Content (TPC)

TPC was determined following the procedure outlined in [16]. In short, 0.2 g of each sample was extracted twice using 10 mL of 70% methanol for 10 min. The resulting extract was centrifuged at 5000 rpm for 10 min, and the supernatant was gathered. After double dilution, 100 µL of the sample was combined with 1 mL of Folin and Ciocalteu’s reagent and 0.8 mL of 7.5% sodium carbonate. The mixture was left in the dark at 25 °C for 1 h, after which the absorbance was recorded at 765 nm. TPC was expressed in micrograms of gallic acid equivalent per gram of wheat bran.

#### 2.6.2. Flavonoid Content (FC)

FC was measured following the procedure outlined in a previous study [29]. Wheat bran was treated with 80% methanol via sonication for 1 h, followed by centrifugation at 5000 rpm for 10 min to obtain the extract. The precipitate was re-extracted, and the supernatants were pooled. Then, 500 μL of the extract was combined with 0.15 mL of 5% sodium nitrite and 2 mL distilled water. After 5 min, 0.15 mL of 10% aluminum chloride was added, and, after another 5 min, 1 mL of sodium hydroxide (1 M) was added. Absorbance was measured at 420 nm after a 15 min reaction. FC was expressed as milligrams of rutin per gram of wheat bran.

#### 2.6.3. Alkylresorcinol Content (ARC)

ARC was determined following the protocol outlined in a previous study [16]. In short, 1 g of wheat bran was extracted with 10 mL of acetone at room temperature for 1 h under continuous stirring. The solution was filtered, and the combined extracts were evaporated at 45 °C under reduced pressure. The dried residue was re-dissolved in methanol. Next, 400 μL of the extract was mixed with 4 mL Fast Blue RR Salt reagent (0.05% Fast Blue RR Salt in 100 mL of 1% acetic acid aqueous solution, combined with methanol at a 1:5 ratio). The absorbance was measured at 480 nm after allowing the reaction to sit at room temperature for 60 min. ARC is expressed as milligrams of olive phenol equivalent (OE) per gram of wheat bran.

#### 2.6.4. pH Value

A total of 1 g of crude fermented wheat bran was combined with 10 mL of distilled water and stirred continuously for 30 min. The pH was then measured using a PHSJ-3F pH meter (INESA Scientific Instrument Co., Ltd., Shanghai, China) [30].

### 2.7. Antioxidant Activity Assays

The fermented wheat bran was extracted using 80% methanol in an ultrasonic bath for 60 min, followed by centrifugation at 5000 rpm for 10 min to create the sample solution. The precipitate was re-extracted several times, and the supernatants were combined to obtain the solution for subsequent analysis of radical scavenging activity [29].

#### 2.7.1. DPPH Radical Scavenging Assay

The DPPH radical scavenging rate was assessed based on the method in [31] with minor adjustments. In short, 2 mL of each sample were combined with 2 mL of 0.1 mM DPPH ethanol solution, followed by a 40 min reaction at room temperature in subdued light after vigorous shaking. The absorbance was then recorded at 517 nm. Distilled water was used as the control instead of the extract. The DPPH radical scavenging rate of wheat bran samples was calculated using the following formula:

(2) $$\mathrm{DPPH}\;\mathrm{radical}\;\mathrm{scavenging}\;\mathrm{rate}\;(\%)=\frac{A\mathrm{control}-A\mathrm{sample}}{A\mathrm{control}}\times 100$$

#### 2.7.2. ABTS Radical Scavenging Assay

The ABTS radical scavenging rate was measured using the method described in [32] with minor adjustments. ABTS working solution was prepared by reacting ABTS (7 mM) with potassium persulfate (2.45 mM) for 12 h in darkness and then diluted with PBS buffer (pH 7.4) until the absorbance at 734 nm reached 0.70–0.8. Each sample (1 mL) was mixed with 1 mL of the ABTS working solution and allowed to react in the dark for 30 min. The absorbance of the test sample (*A*_2_) was recorded at 734 nm. *A*_1_ was the absorbance of the methanol solution used in place of the extract, and *A*_0_ was the absorbance of the methanol solution with the extract. The ABTS radical scavenging rate was calculated using the following formula:

(3) $$\mathrm{ABTS}\;\mathrm{scavenging}\;\mathrm{rate}\;(\%)=(1-\frac{A_{2}-A_{0}}{A_{1}})\times 100$$

#### 2.7.3. Hydroxyl Radical Scavenging Assay

The hydroxyl radical scavenging rate was evaluated using the method described in [33] with minor modifications. In short, 2 mL of the sample solution was combined with 2 mL of FeSO_4_ solution (6 mM) and 2 mL of H_2_O_2_ solution (6 mM). After allowing the mixture to stand at room temperature for 10 min, 2 mL of salicylic acid–ethanol solution (6 mM) was added, and the absorbance (*Ai*) at 517 nm was measured after a 15 min reaction at 37 °C. Distilled water was used in place of the salicylic acid–ethanol solution and H_2_O_2_ solution to measure absorbance (*Aj*) following the same procedure; similarly, distilled water replaced the sample solution to obtain absorbance (*A_0_*). The hydroxyl radical scavenging rate was calculated using the following equation:

(4) $$\mathrm{Hydroxyl}\;\mathrm{radical}\;\mathrm{scavenging}\;\mathrm{rate}\;(\%)=(1-\frac{A_{i}-A_{j}}{A_{0}})\times 100$$

#### 2.7.4. Reducing Ability Assay

The reducing ability (RA) was assessed using the method in [34]. In brief, 1 mL of the sample was mixed with 2.5 mL of phosphate buffer (200 mM, pH 6.6) and 2.5 mL of 1% potassium ferricyanide. After standing at 50 °C for 20 min, 2.5 mL of 10% trichloroacetic acid was added, followed by centrifugation at 3000 rpm for 10 min. The supernatant (2.5 mL) was then combined with 2.5 mL of distilled water and 0.5 mL of 0.1% ferric chloride solution. The absorbance at 700 nm was measured to quantify the reducing ability of the wheat bran sample.

### 2.8. Statistical Analysis

All measurements were conducted in triplicate, and the results were presented as mean values ± standard deviation. Statistical significance was evaluated through one-way ANOVA, followed by Dunnett’s multiple comparisons test using SPSS version 22. Differences were considered statistically significant at *p* ˂ 0.05.

## 3. Results and Discussion

### 3.1. Extraction Yields of SDF from Wheat Bran Under Different Fermentation Conditions

**Figure 1:**
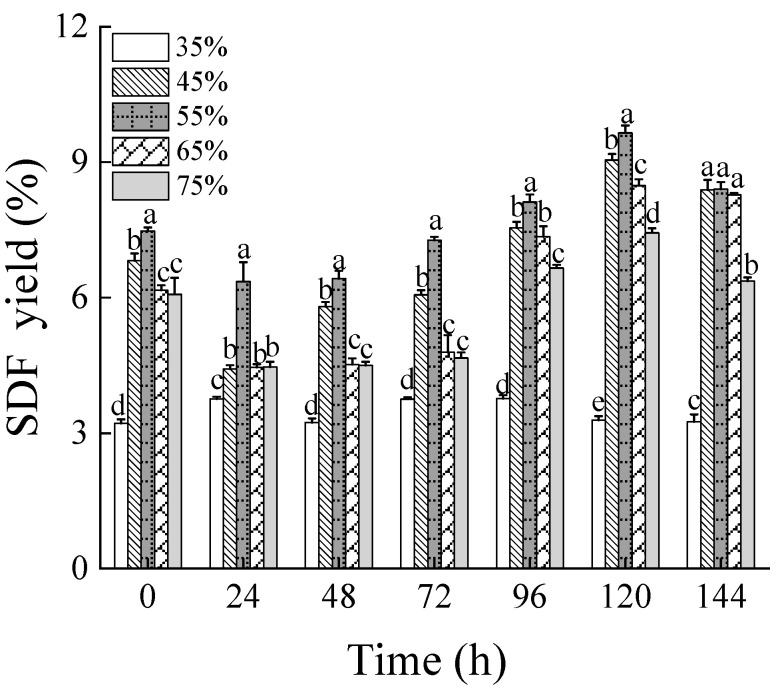
The SDF yield of wheat bran during solid-state fermentation by *Rhizopus oryzae* with different moisture contents and fermentation times. Different superscript letters indicate a significant difference (*p* < 0.05).

Figure 1 shows the yields of soluble dietary fiber (SDF) from wheat bran subjected to solid-state fermentation under different moisture levels of 35%, 45%, 55%, 65%, and 75%, over fermentation times ranging from 0 to 144 h. After fermenting wheat bran at 55% moisture content for 120 h, the maximum SDF yield reached 9.65%, representing an increase of 2.7 times compared to non-treated wheat bran (3.63%). With the rise in substrate moisture levels and extended fermentation duration, the SDF yields first increased and then decreased. The growth and propagation of filamentous fungi rely heavily on the initial moisture levels, particularly in static environments [35]. For *Rhizopus oryzae,* excessive moisture inhibits mycelial growth and reduces substrate porosity, which impedes gas exchange. Conversely, insufficient moisture fails to meet the growth requirements of the mycelium, adversely affecting fermentation outcomes [36]. Thus, the SDF yield first increased and then decreased with both rising moisture content and extended fermentation periods. Furthermore, the SDF yield variably increased following high-pressure sterilization of wheat bran at different moisture content levels, suggesting that hydrothermal treatment can enhance cell wall cleavage and release more SDF [25].

### 3.2. Analysis of Enzyme Activity During Fermentation

**Figure 2:**
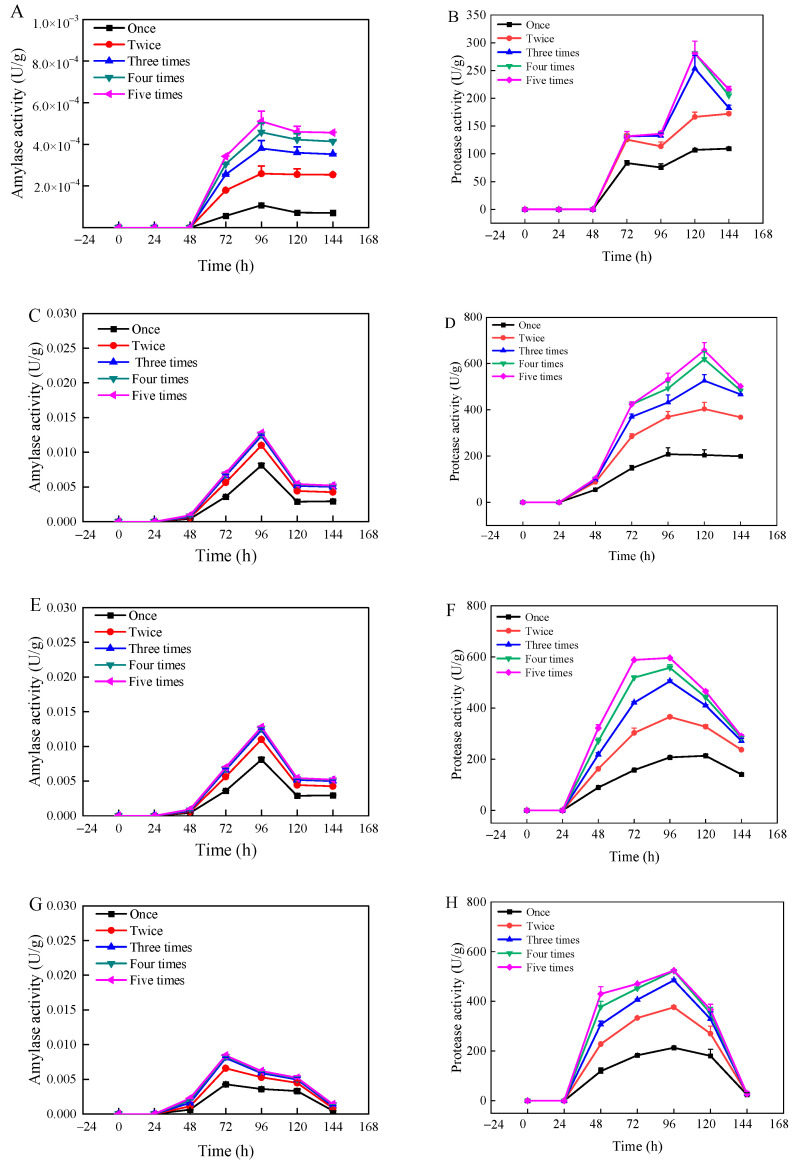

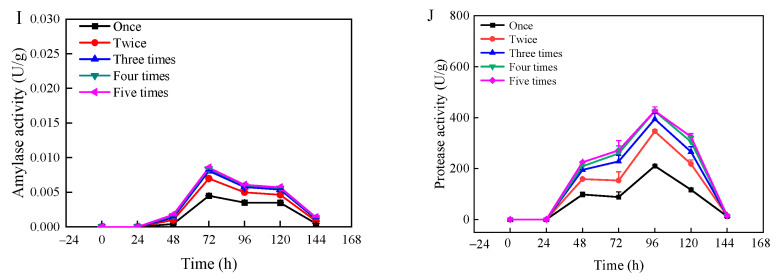
Changes in amylase activity (**A**,**C**,**E**,**G**,**I**) and protease activity (**B**,**D**,**F**,**H**,**J**) of wheat bran during solid-state fermentation by *Rhizopus oryzae* with different moisture contents and fermentation times.

The changes in amylase and protease activities with moisture content and fermentation time are shown in Figure 2. The enzyme activities varied significantly with changes in substrate moisture content and fermentation time, as illustrated for amylase activity (A, C, E, G, I) and protease activity (B, D, F, H, J). At a moisture content of 35%, both protease and amylase activities were relatively low, with the highest recorded amylase activity at 5.1 × 10^−4^ U/g and protease activity at 281.2 U/g. This low activity level could be attributed to the insufficient moisture content, which stunts the growth of *Rhizopus oryzae* and its enzymatic productivity. Conversely, when the moisture content ranged from 45% to 55%, there was a significant increase in both protease and amylase activities. Notably, amylase activity peaked at 55% moisture content, while protease activity was highest at 45% moisture content. Afterwards, the enzymatic activities decreased with increasing substrate moisture content, likely due to excessive moisture hampering microbial growth and enzyme production. During the early phase of fermentation (2 d), only a small number of enzymes were produced, likely because the strain needed time to adjust to the new environment and had not yet developed enzyme-producing capabilities [37]. Amylase activity reached its maximum at 96 h for moisture contents between 35% and 55%, and at 72 h for moisture contents between 65% and 75%. Protease activity peaked at 120 h for moisture contents between 35% and 45%, and at 96 h for moisture contents between 55% and 75%, after which enzyme activities began to decrease. This decline could be attributed to the differential impact of moisture content on the growth dynamics of *Rhizopus oryzae* across varying fermentation durations, affecting enzyme synthesis capabilities. Figure 2 illustrated that the amylase activity had no significant change after three times of extraction, and the protease activity tended to be stable after four times of extraction. This stabilization likely results from the complete extraction of enzyme activity generated during the fermentation process.

Table S4 presents the changes in core temperature of wheat bran during fermentation under various substrate moisture content and fermentation time. At 35% moisture content, the central temperature showed negligible fluctuations and remained relatively low, correlating with the lowest enzyme activity levels observed. By contrast, for moisture contents ranging from 45% to 75%, the central temperature varied, reaching its apex at 55% moisture content. After 96 h of fermentation, the central temperature stabilized at 28.90 ± 0.36 °C, coinciding with vigorous fungal growth. Optimal water activity for most molds ranges from 0.80 to 0.94; thus, growth is inhibited when water activity falls below 0.60 [38]. Table S5 depicts the temporal changes in water activity of fermented wheat bran across different substrate moisture content. At 45% and 55% moisture contents, the water activity predominantly fell within the range conducive to microbial growth [39]. As shown in Figure S3, at 35% moisture content, adsorbed water is much higher than that of capillary water, and the peak time also occurred significantly later than for other groups. For moisture content between 45% and 75%, capillary water predominated over adsorbed water. As the solid-state fermentation of wheat bran progresses, water evaporation and microbial consumption from the substrate are mainly sourced from capillary water. From 72 to 144 h of fermentation, the overall level of capillary water exhibited a declining trend. Notably, at 96 h, the proportion of capillary water significantly diminished, likely due to accelerated biomass growth of *Rhizopus oryzae* and consequent increased consumption of capillary water. Throughout the measurement process, when the substrate had a moisture content of 55% and fermentation lasted 96 h, the amylase activity peaked at 0.013 U/g. When the moisture content was 45% and fermentation extended to 120 h, protease activity reached its highest value of 656.9 U/g. When the moisture content was 45% and fermentation extended to 120 h, protease activity reached its highest value of 656.9 U/g, exhibiting significant higher protease activity compared to the investigation from [21], where most of protease was recovered 67.8 U/g in three extractions. The results suggested that fungal-based solid-state fermentation might be a promising technology for protease production.

### 3.3. Morphology

**Figure 3:**
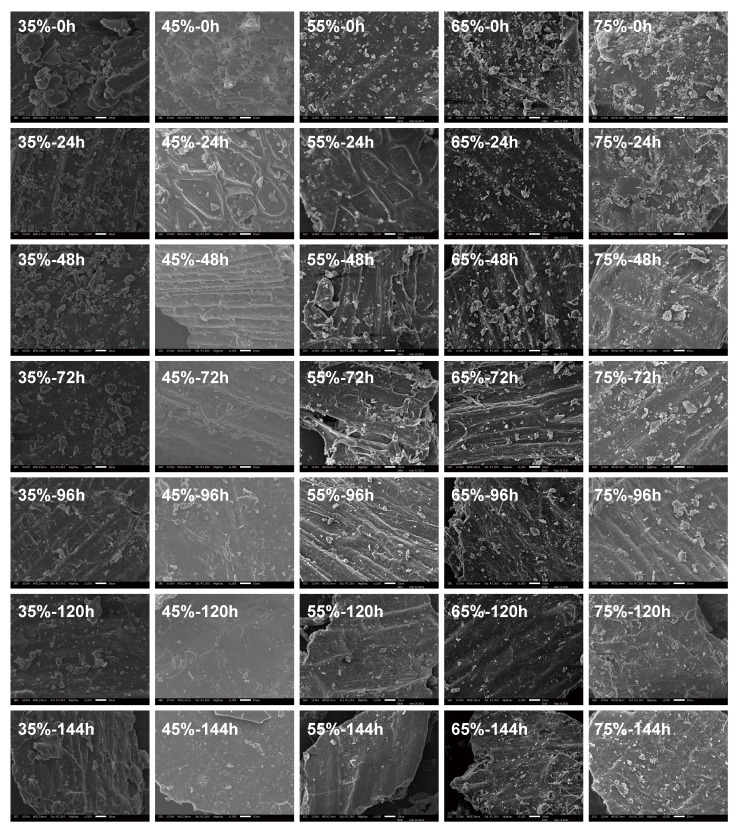
Morphology of wheat bran during solid-state fermentation by *Rhizopus oryzae* with different moisture content and fermentation time.

The morphologies of fermented wheat brans under varying substrate moisture content and fermentation time were examined by SEM (Figure 3). Additionally, Figure S4 shows the morphology of raw wheat bran, where starch granules in the aleurone layer are distinctly visible. After sterilization, the aleurone layer is destroyed, and the starch granules are obviously reduced. The extent of degradation varies with substrate moisture content; the most significant degradation occurs at 55% moisture, as shown in Figure 3. At 45% moisture, the structural changes in wheat bran are akin to those at 55%, with visible transverse and tubular cells. Conversely, at 75% moisture, starch granules in the aleurone layer are still observable after 144 h of fermentation, indicating lesser degradation, which is minimal at 35% moisture content. As fermentation advances, the starch granules in the aleurone layer decrease, exposing the tubular cells on the bran surface, leaving only the smooth outer layer visible [40]. Structurally, wheat bran consists of three layers: the outer skin, the middle, and the aleurone layers [41]. Previous research using *Lactobacillus plantarum* and *Saccharomyces cerevisiae* together showed similar trends in starch granule degradation and utilization [42]. From the above analysis, amylase and protease can be produced during the SSF of wheat bran by *Rhizopus oryzae*, and amylase can degrade residues and starch in wheat bran, as shown in Figure 3. With the extension of fermentation time, starch particles on the aleurone layer gradually decrease. Protease can hydrolyze the structural proteins in the cell wall to degrade the cell wall. Especially in the middle and late stages of fermentation, protease activity reaches its highest point and significantly promotes the degradation of the cell wall. Therefore, the SSF of wheat bran by *Rhizopus oryzae* can degrade part of the aleurone layer and the middle layer of wheat bran.

### 3.4. Physicochemical Properties of Wheat Bran

**Figure 4:**
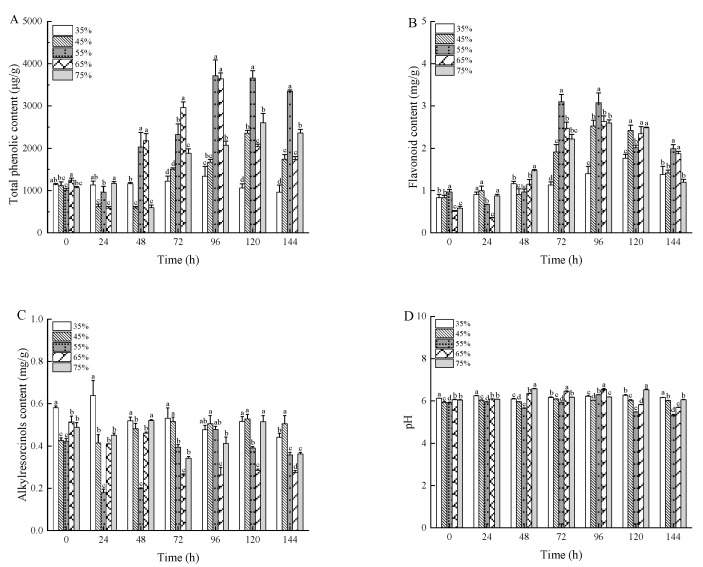
The total phenolic content (**A**), flavonoid content (**B**), alkylresorcinols content (**C**), and pH (**D**) of wheat bran during solid-state fermentation by *Rhizopus oryzae* with different moisture content and fermentation times. Different superscript letters indicate a significant difference (*p* < 0.05).

The total phenolic content (TPC) peaked at 3713.64 μg/g when the substrate had 55% moisture content and fermentation lasted 96 h, which is 3.27 times higher than in unfermented wheat bran, as depicted in Figure 4A. TPC increased during the early phase of fermentation but slightly declined toward the end. As noted by [43], solid-state fermentation aids in converting bound phenolics to free phenolics, improving their release and bioavailability. Figure 4B highlights the changes in flavonoid content (FC) during fermentation. The largest increase in FC was observed when the wheat bran substrate had a moisture content of 55%. Initially, there were minimal changes in FC, but a sharp rise occurred in the later stages of fermentation. This study showed that the flavonoid content in wheat bran increased significantly after fermentation, with FC reaching the peak 3.10 mg/g at 72 h of solid-state fermentation, a substantial increase from the 0.72 mg/g in unfermented wheat bran. As in previous studies, fermentation enhanced the release of flavonoids and other bioactive compounds in wheat bran, likely due to the increased specific surface area of the bran [29]. In addition, the release of phenolic and flavonoid compounds was enhanced due to extracellular enzymatic hydrolysis activity of fungi in the wheat bran. During SSF, various hydrolysis enzymes might be produced such as *α*-amylase, proteases, hemicellulase, phenolic esterase, and lipases that could break down the phenolic compounds attached to cell wall components, such as cellulose, hemicellulose, and proteins in conjugated forms [44,45,46].

This study examined the changes in alkylresorcinols content (ARC) and the pH value of wheat bran during fermentation. As shown in Figure 4C,D, ARC in unfermented wheat bran measured 0.45 mg/g, with a pH of 6.07. After sterilization and fermentation, samples with lower pH showed reduced ARC levels. This decrease in ARC is likely due to acid generation during fermentation, leading to acidification and a corresponding reduction in alkylresorcinols. Throughout fermentation, the variations in ARC were minimal, likely because the pH remained relatively stable, ranging from 5.23 to 6.58. These results align with previous studies [47,48], which also observed only slight changes in alkylresorcinols content.

### 3.5. Changes in Antioxidant Capacity

This study conducted different antioxidant assays including DPPH, ABTS, hydroxyl radical rate, and reducing ability to examine distinct radical-scavenging mechanisms and provide information about different antioxidant molecules. The antioxidant activities, determined by DPPH, ABTS, and hydroxyl radical tests, were expressed as a percentage of radical-scavenging capacity. Meanwhile, the reducing ability was indicated by absorbance values, reflecting the antioxidant potential of the product.

**Figure 5:**
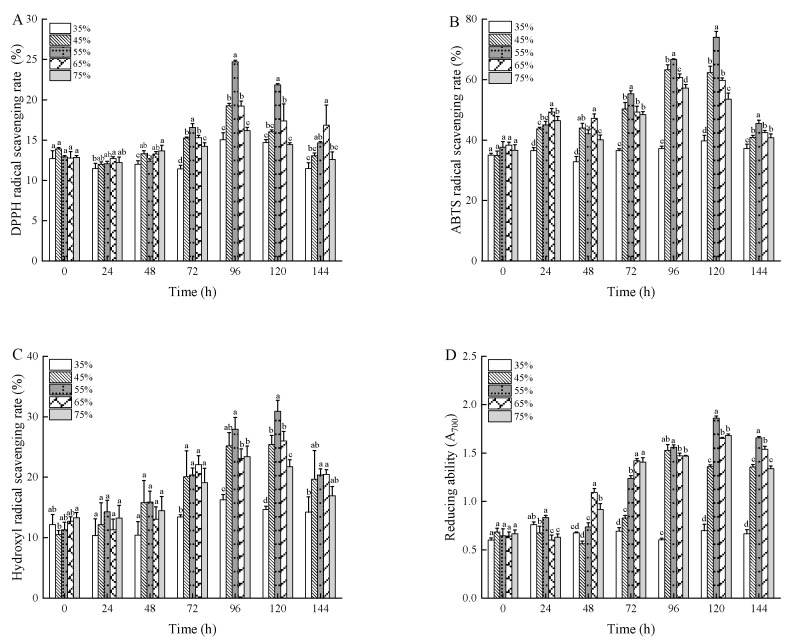
The DPPH radical-scavenging rate (**A**), ABTS radical-scavenging rate (**B**), hydroxyl radical-scavenging rate (**C**), and reducing ability (**D**) of wheat bran during solid-state fermentation by *Rhizopus oryzae* with different moisture content and fermentation times. Different superscript letters indicate a significant difference (*p* < 0.05).

Figure 5 shows that autoclaving under various conditions enhanced the antioxidant activity of wheat bran extracts. This improvement could be due to thermal processes that either boost antioxidant properties or result in the creation of new antioxidant compounds, such as Maillard reaction products. It is well documented that phenolics and flavonoids play crucial roles in terminating free radical reactions, exhibiting significant free-radical-scavenging effects [49]. Furthermore, phenolic acids are reported to possess stronger antioxidant capacities compared to flavonoids. Additionally, regardless of the increases in moisture content or fermentation time of the wheat bran substrate, the antioxidant activity exhibited a pattern of initial increase followed by a subsequent decrease.

As shown in Figure 5A, the antioxidant activity during wheat bran fermentation reveals that unfermented wheat bran exhibited a 12.23% scavenging ability against DPPH radicals. This increased to a peak of 24.73% when the moisture content was kept at 55% and fermentation extended to 96 h. At this point, the phenolic content of the fermented wheat bran was also at its highest, supporting the link between DPPH radical scavenging rate and polyphenol levels. Previous studies on fermented plant antioxidants suggest a direct correlation between antioxidant activity and free polyphenol content. The efficiency of phenols in neutralizing DPPH radicals is largely due to their hydrogen-donating ability [50].

As described in the literature [51], ABTS radicals are quenched via electron or hydrogen atom transfer. Figure 5B shows that the ABTS radical scavenging ability of unfermented wheat bran started at 30.32% and increased significantly to a peak of 74.04% when the substrate had a moisture content of 55% and was fermented for 120 h. Notably, the ability to scavenge ABTS radicals in both unfermented and fermented wheat bran exceeded that of DPPH radicals. Hydroxyl radicals, mainly produced by the Fenton reaction in the presence of transition metals, can react with biomolecules, causing significant tissue damage and cell death [52].

As shown in Figure 5C, the hydroxyl radical-scavenging capacity of unfermented wheat bran was initially 11.76%. This capacity increased to a maximum of 30.91% under conditions of 55% moisture content and 120 h of fermentation time, which represents an enhancement of 19.15% over the raw wheat bran. This improvement may be attributed to certain components in the wheat bran fermentation products that have the ability to chelate metal ions, thereby inhibiting the production of hydroxyl radicals [52].

The reducing ability assay, which evaluates electron donation potential, is a key marker of antioxidant capacity; substances with lower single-electron redox potential exhibit stronger reducing ability and antioxidant effects. Figure 5D shows that unfermented wheat bran had a reducing ability absorbance of 0.461. After 120 h of fermentation at 55% moisture content, this value increased to 1.860, representing a rise of 1.399 compared to the original. The absorbance values, influenced by moisture content and fermentation time, followed a pattern of rising initially and then declining, consistent with the trends observed in antioxidant assays.

In short, these findings highlight that *Rhizopus oryzae* solid-state fermentation significantly boosts the antioxidant capacity of wheat bran. During solid-state fermentation, the activities of protease and amylase facilitate the release of bound phenolic and flavonoid compounds from the wheat bran cell wall. These free phenolics and flavonoids act as key antioxidant molecules by donating hydrogen atoms or electrons to neutralize free radicals, thereby enhancing antioxidant capacity [53]. Secondary metabolites produced by *Rhizopus oryzae* during fermentation, such as organic acids and certain phenolic compounds, may also contribute to the observed antioxidant capacity [54]. Fungal biomass itself may contain antioxidant molecules such as ergosterol and fungal polysaccharides. These compounds can influence the results of antioxidant assays [55]. Furthermore, our study observed a positive correlation between antioxidant capacity such as DPPH radical scavenging and protease/amylase activities. This relationship can be explained by the fact that protease and amylase hydrolyze proteins and starch, releasing bound antioxidant molecules. Higher enzymatic activity often coincides with the active metabolic phase of fungi, which may further enhance the accumulation of antioxidant molecules.

### 3.6. Correlation Analysis

**Figure 6:**
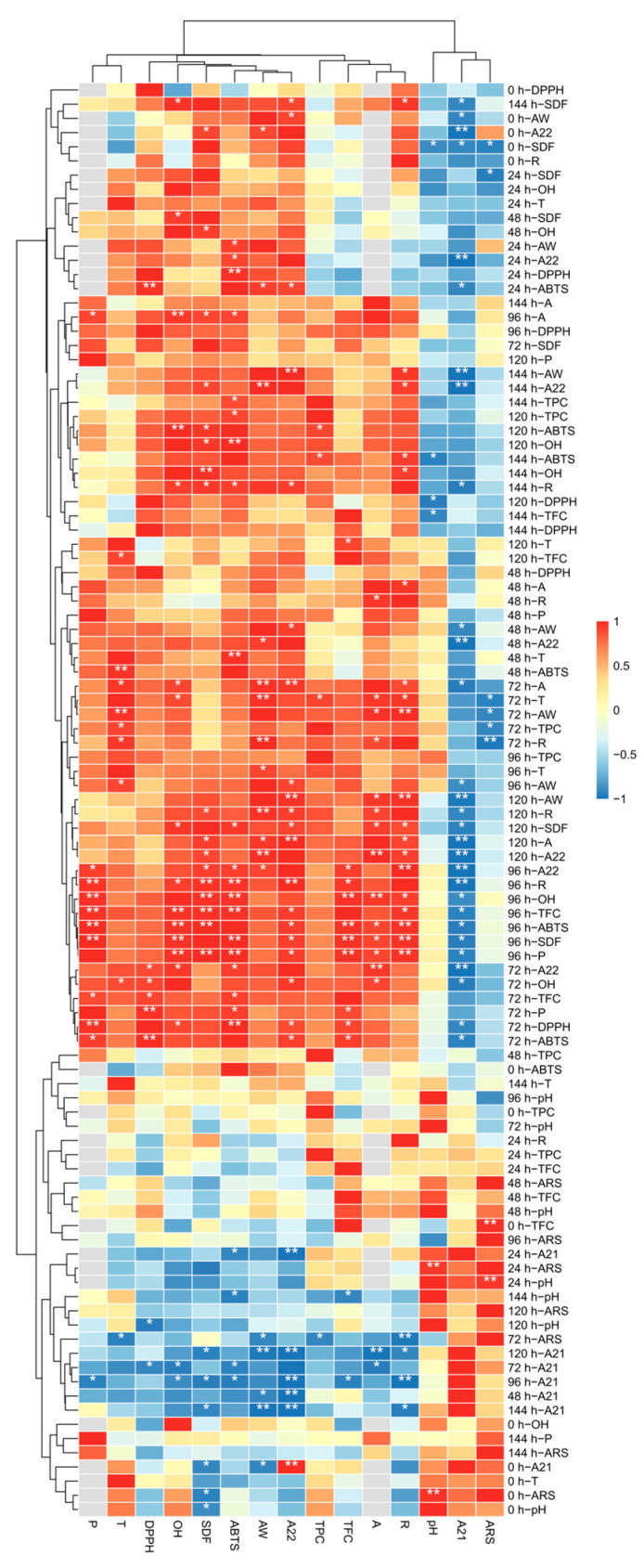
Under the condition of constant fermentation time, the correlation under the influence of moisture content. A, amylase activity, P, protease activity, R, reducing power; “*”, indicates the correlation is significant at 0.05; “**”, indicates the correlation is significant at 0.01.

The correlation analysis among above various indicators were conducted with different moisture contents and fermentation times (Figure 6, Tables S6 and S7). Since moisture content has an important effect on fungal growth [56], we focused on the correlation analysis between various factors under different water contents. The moisture content not only affected microbial metabolism [56] but also correlated with substrate water activity as illustrated in Figure 6, which in turn had a significant effect on enzyme activity, and enzyme activity was positively correlated with antioxidant capacity, which was consistent with the results of our above experimental analysis. At a fermentation time of 96 h, both amylase and protease activities showed a strong positive correlation with the soluble dietary fiber (SDF) extraction yield, exceeding a correlation coefficient of 0.9, with protease activity demonstrating an even higher correlation of 0.999. When the fermentation duration was extended to 120 h, the SDF extraction yield remained positively correlated with both amylase and protease activities, although the correlation with amylase activity was higher at 0.934. Water activity positively influenced the SDF extraction yield and was correlated with protease activity at 96 h and amylase activity at 120 h. Additionally, enzymatic activities showed a positive correlation with total phenolic (TPC) and flavonoid (TFC) content, both of which were associated with antioxidant activity. These results suggest that water activity influences not only SDF extraction but also enzymatic performance, which subsequently affects TPC and TFC levels, thereby altering antioxidant capacity.

## 4. Conclusions

Wheat bran, a key by-product of wheat processing, is a valuable source of dietary fiber. This study used low-cost wheat bran as a substrate and applied *Rhizopus oryzae* for solid-state fermentation to boost SDF content. The enzymatic activities of amylase and protease, alongside the physicochemical properties of wheat bran, were evaluated throughout fermentation. Five substrate moisture levels (35%, 45%, 55%, 65%, and 75%) were tested, with fermentation times ranging from 0 to 144 h. Amylase production peaked at 55% moisture content, which was linked to the highest degree of wheat bran degradation, improved physicochemical characteristics, and elevated phenol and flavonoid levels, resulting in enhanced antioxidant activity and the highest SDF extraction rate. In summary, we conducted a cumulative enzyme activity determination method to quantify total enzyme activity including free and absorbent enzyme activities during solid-state fermentation of wheat bran by *Rhizopus oryzae*. We found that the amylase activity had no significant change after three times of extraction, and the protease activity tended to be stable after four times of extraction. The SDF yield, physicochemical properties (water activity, central temperature, moisture composition, total phenolic content, flavonoid content, alkylresorcinols content, and pH value), antioxidant activities (DPPH, ABTS, hydroxyl radical scavenging rates, and reducing ability), and microstructure were analyzed throughout the fermentation process. Moreover, the correlation analysis among the above various indicators was conducted with different moisture content and fermentation times. The methodologies in this study and its prospective results could lay theoretical support for the understanding of the mechanisms for regulating high solid fermentation efficiency and the application of high-quality dietary fiber from wheat bran.

## Data Availability

The original contributions presented in the study are included in the article/Supplementary Material, further inquiries can be directed to the corresponding author.

## Supplementary Materials

The following supporting information can be downloaded at https://www.mdpi.com/article/10.3390/foods13243998/s1, Table S1: Chemical composition of wheat bran; Figure S1: Effect of water content of media on the extraction of SDF (A), effect of inoculum’s volume on the extraction of SDF (B), effect of particle size on the extraction of SDF (C), effect of fermentation temperature on the extraction of SDF (D), effect of fermentation time on the extraction of SDF (E); Table S2: The design and results of response surface experiments of SDF; Table S3: Variance analysis of regression equation; Figure S2: Influence of substrate moisture content and inoculum (A); influence of substrate moisture content and particle size (B); influence of substrate moisture content and fermentation time (C); influence of particle size and inoculum (D); influence of fermentation time and inoculum (E), influence of fermentation time and particle size (F); Table S4: Changes in central temperature of fermented wheat bran with time under different substrate moisture content; Table S5: Changes in water activity of fermented wheat bran with different substrate moisture content; Figure S3: Effect of different substrate moisture content on moisture relaxation time T2 of wheat bran; Figure S4: Observation of the morphology of raw wheat bran; Table S6: Correlation under the influence of moisture content and fermentation time; Table S7: Under the condition of constant moisture content, the correlation under the influence of fermentation time.
