# Supplementary material for: Correlation Analyses of Amylase and Protease Activities and Physicochemical Properties of Wheat Bran During Solid-State Fermentation
Source: Foods. 2024 Dec 11;13(24):3998. doi: 10.3390/foods13243998 (PMC11675429; doi:10.3390/foods13243998)
Supplement: Supplementary file 1 [file foods-13-03998-s001.zip › Supplementary Material.pdf]

Supplementary Material

Table S1. The chemical compositions of wheat bran.

Chemical composition	Content (%)
Moisture	13.34±0.14
Ash	6.00±0.16
Lipid	3.00±0.01
Protein	14.64±0.46
starch	16.29±0.44
SDF	2.75±0.12
IDF	45.71±0.08
TDF	48.46±0.20

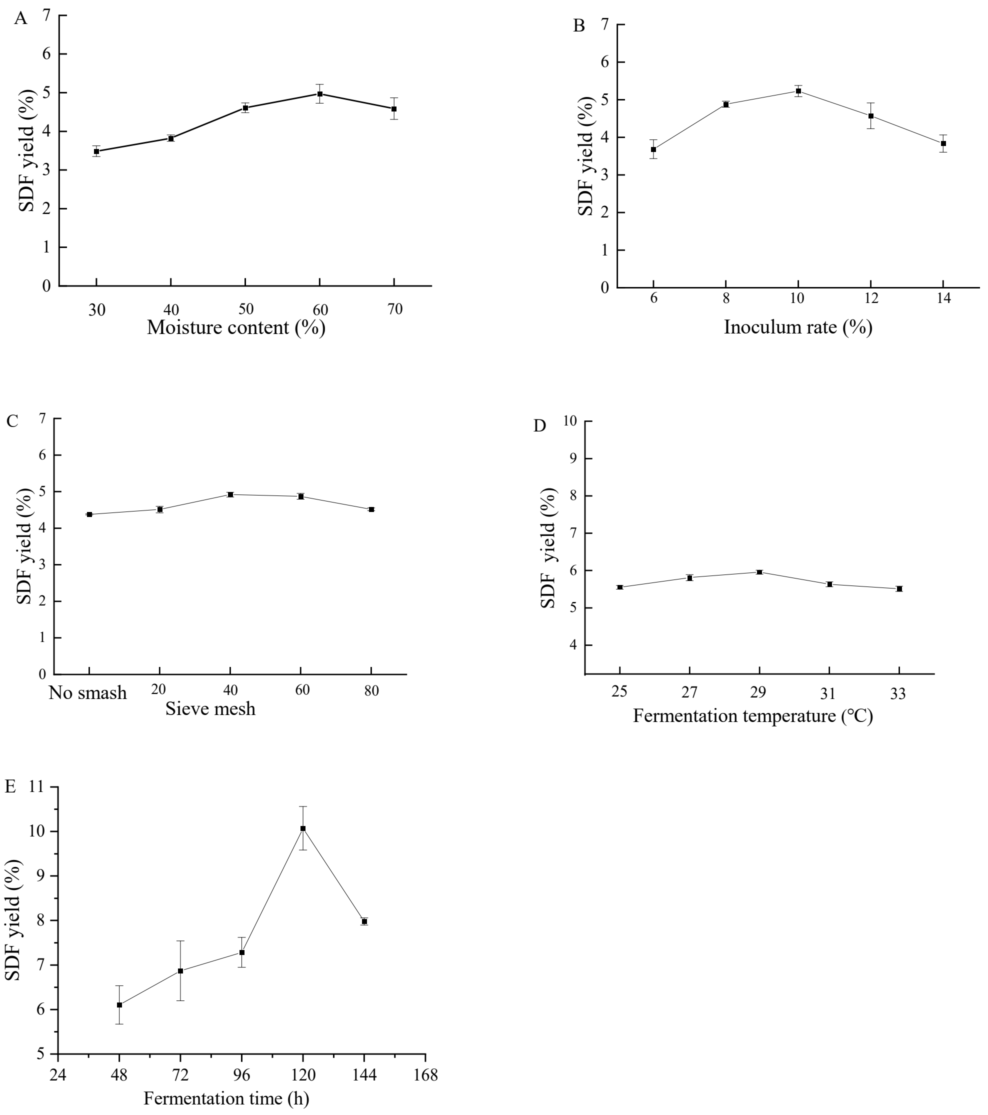

Figure S1. Effect of moisture content (A), inoculum rate (B), sieve mesh (C), fermentation temperature (D), and fermentation time (E) on SDF yield.

Table S2. The design and results of response surface experiments.

Number	Moisture content	Inoculum rate	Sieve mesh	Fermentation time	SDF yield
1	0	-1	-1	0	9.39
2	0	0	0	0	9.47
3	0	0	1	1	8.78
4	-1	0	0	-1	7.86
5	-1	0	1	0	9.30
6	0	1	-1	0	9.04
7	0	-1	0	1	8.35
8	1	0	0	-1	6.98
9	-1	0	0	1	8.59
10	0	0	0	0	9.45
11	0	0	-1	-1	7.83
12	-1	-1	0	0	8.39
13	0	0	1	-1	7.15
14	0	-1	0	-1	7.64
15	0	0	-1	1	8.43
16	1	0	0	1	8.42
17	0	1	1	0	9.3
18	0	0	0	0	9.45
19	0	1	0	1	8.88
20	1	0	-1	0	8.79
21	0	-1	1	0	8.77
22	0	1	0	-1	7.61
23	1	1	0	0	7.68
24	-1	0	-1	0	9.45
25	1	0	1	0	9.67
26	0	0	0	0	9.47
27	0	0	0	0	9.91
28	-1	1	0	0	9.26
29	1	-1	0	0	8.48

Table S3. Variance analysis of regression equation.

Variance	Sum of square	Degrees of freedom	Mean square	F value	P	Significant
Source	SS	df	MS			
Total model	15.79	14	1.13	20.84	<0.0001	**
A	1.22	1	1.22	22.59	0.0003	**
B	0.047	1	0.047	0.87	0.3678	
C	0.077	1	0.077	1.42	0.2533	
D	3.39	1	3.39	62.68	<0.0001	**
AB	0.70	1	0.70	12.88	0.0030	**
AC	0.000225	1	0.000225	0.004158	0.9495	
AD	013	1	013	2.33	0.1493	
BC	0.19	1	0.19	3.58	0.0794	
BD	0.078	1	0.078	1.45	0.2487	
CD	0.27	1	0.27	4.9	0.0439	*
A^2	1.63	1	1.63	30.12	<0.0001	**
B^2	0.97	1	0.97	17.88	0.0008	**
C^2	0.097	1	0.097	1.8	0.2012	
D^2	8.88	1	8.88	164.08	<0.0001	**
Residual	0.76	14	0.054			
Lack of fit error	0.6	10	0.06	1.47	0.3799	-
Random error	0.16	4	0.041			
sum	16.54	28				

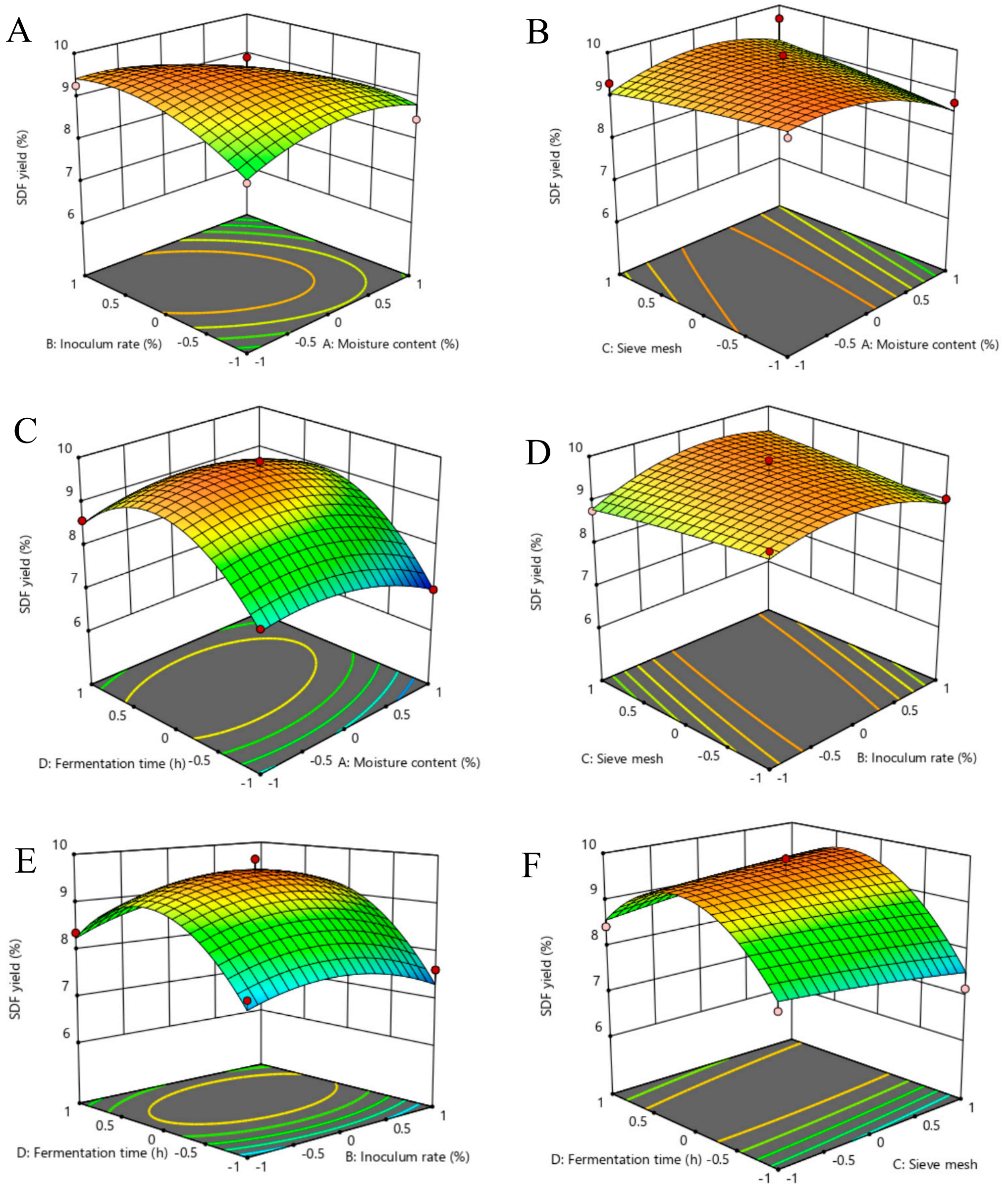

Figure S2. Influence of moisture content and inoculum rate (A), influence of moisture content and sieve mesh (B), influence of moisture content and fermentation time (C); influence of sieve mesh and inoculum rate (D); influence of fermentation time and inoculum rate (E), influence of fermentation time and sieve mesh (F) on SDF yield.

Determination of central temperature

The fermentation process of wheat bran by *Rhizopus oryzae* was monitored with food-grade thermometer probe at different moisture contents of 35%, 45%, 55%, 65%, and 75% for 144 h. The central temperature data were measured three times as parallel.

Table S4. Changes in the central temperature of fermented wheat bran with different moisture contents and fermentation times.

Moisture content	0 h	24 h	48 h	72 h	96 h	120 h	144 h
35%	25.30 ± 0.20^{a}	26.03 ± 0.15^{c}	27.10 ± 0.26^{c}	26.13 ± 0.25^{c}	26.80 ± 0.17^{b}	26.43 ± 0.21^{c}	25.23 ± 0.25^{b}
45%	25.17 ± 0.06^{a}	26.13 ± 0.25^{c}	28.37 ± 0.21^{ab}	27.10 ± 0.20^{b}	27.30 ± 0.20^{b}	27.63 ± 0.25^{a}	26.60 ± 0.00^{a}
55%	24.90 ± 0.36^{a}	27.67 ± 0.15^{a}	27.80 ± 0.60^{b}	27.87 ± 0.60^{a}	28.90 ± 0.36^{a}	26.57 ± 0.58^{c}	24.97 ± 0.42^{b}
65%	25.07 ± 0.25^{a}	27.23 ± 0.23^{b}	28.63 ± 0.50^{a}	28.57 ± 0.29^{a}	28.77 ± 0.42^{a}	27.10 ± 0.17^{b}	26.57 ± 0.40^{a}
75%	25.17 ± 0.06^{a}	27.83 ± 0.32^{a}	27.93 ± 0.30^{ab}	28.03 ± 0.40^{a}	28.53 ± 0.64^{a}	27.23 ± 0.23^{b}	26.93 ± 0.15^{a}

Different superscript letters in the same column indicate a significant difference ($p < 0.05$).

Determination of water activity

The water activity of fermented wheat bran by *Rhizopus oryzae* was monitored with automatic water activity detector at different moisture contents of 35%, 45%, 55%, 65%, and 75% for 144 h. The water activity data were measured three times as parallel.

Table S5. Changes in the water activity of fermented wheat bran with different moisture contents and fermentation times.

Moisture content	0 h	24 h	48 h	72 h	96 h	120 h	144 h
35%	0.868±0.01^{c}	0.901±0.01^{d}	0.905±0.01^{d}	0.877±0.02^{c}	0.865±0.02^{d}	0.759±0.07^{b}	0.748±0.07^{b}
45%	0.916±0.01^{b}	0.921±0.01^{c}	0.931±0.01^{c}	0.906±0.01^{b}	0.912±0.00^{c}	0.905±0.02^{a}	0.911±0.01^{a}
55%	0.931±0.01^{b}	0.937±0.00^{bc}	0.939±0.00^{b}	0.931±0.00^{a}	0.938±0.00^{bc}	0.955±0.00^{a}	0.947±0.01^{a}
65%	0.949±0.00^{a}	0.947±0.01^{ab}	0.946±0.00^{a}	0.950±0.00^{a}	0.951±0.00^{ab}	0.957±0.00^{a}	0.955±0.00^{a}
75%	0.955±0.00^{a}	0.953±0.00c^{a}	0.950±0.00^{a}	0.950±0.00^{a}	0.959±0.00^{a}	0.963±0.00^{a}	0.958±0.01^{a}

Different superscript letters in the same column indicate a significant difference ($p < 0.05$).

Low-field NMR analysis

Raw and fermented wheat bran (0.6 g) was subjected to low-field NMR experiment with a scan of CPMG pulse sequence. The sampling point TD was 25024, sampling frequency SW was 250.00 kHz, sampling interval time TW was 2000 ms, and cumulative frequency NS was 4. The spin-spin relaxation time was recorded as T2, and repeatedly measured three times as parallel.

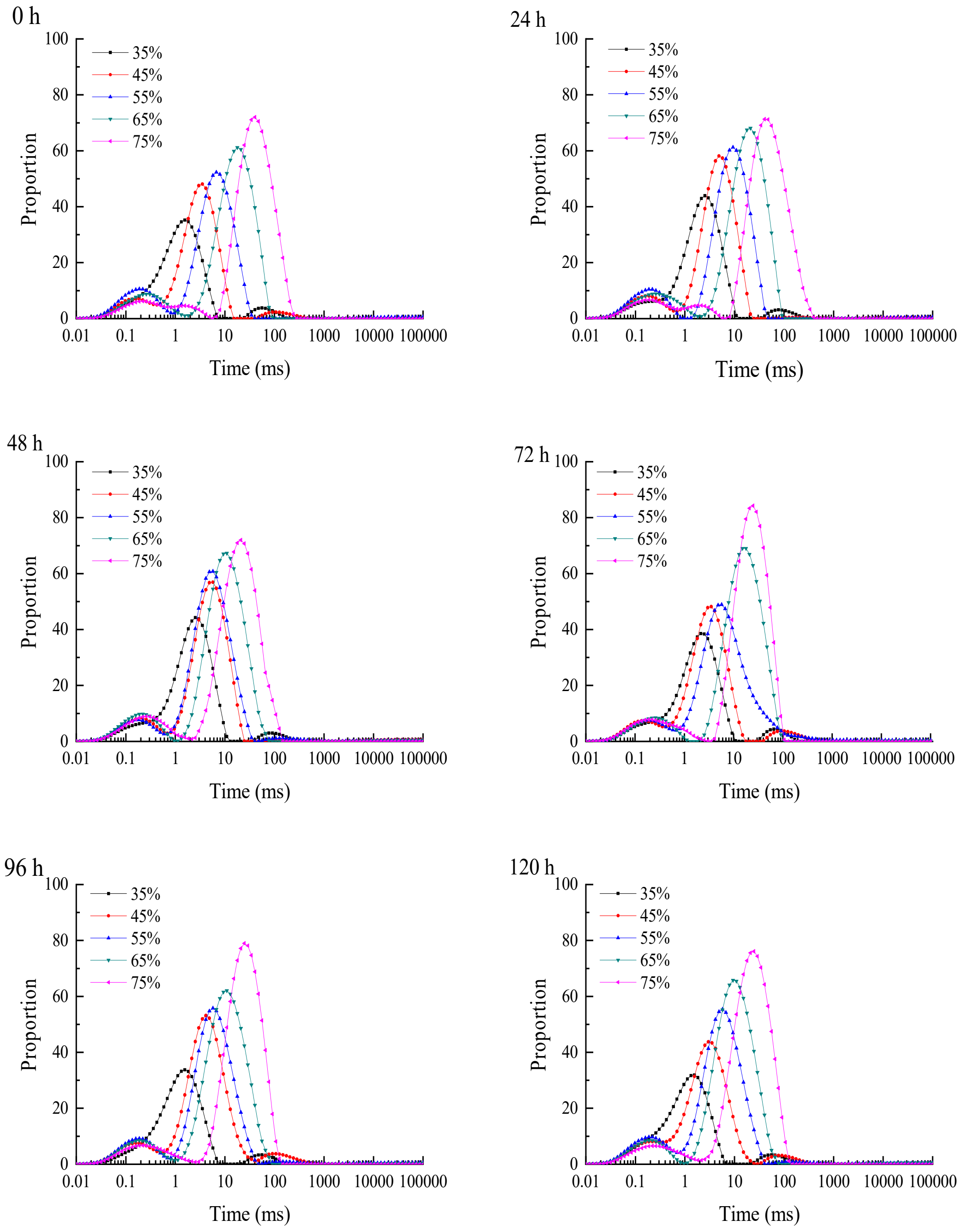

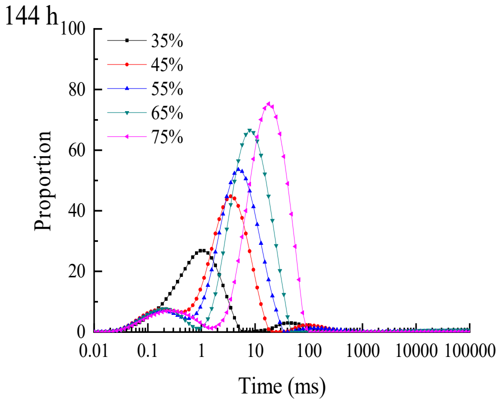

Figure S3. The moisture relaxation time T2 of fermented wheat bran with different moisture contents and fermentation times.

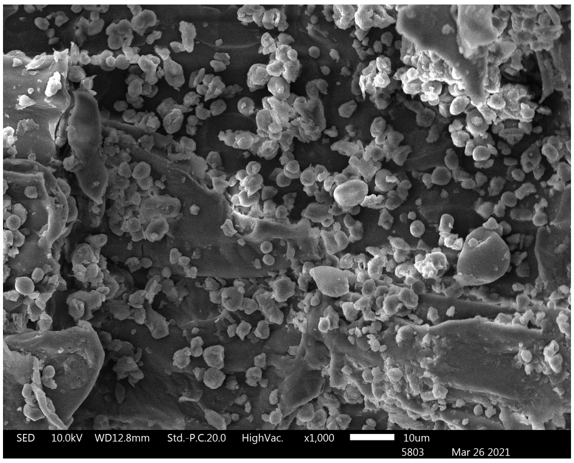

Figure S4. The morphology of raw wheat bran.

Table S6. Correlation under the influence of moisture content and fermentation time.

Indicators	SDF	A	P	T	A_W	A_{21}	A_{22}	TPC	TFC	ARS	pH	DPPH	ABTS	OH	R
SDF	1	0.355*	0.357*	-0.121	0.631**	-0.756**	0.753**	0.443**	0.271	-0.180	-0.545**	0.523**	0.523**	0.624**	0.607**
A	0.355*	1	0.788**	0.440**	0.339	-0.403*	0.398*	0.588**	0.836**	0.049	0.161	0.708**	0.696**	0.775**	0.723**
P	0.357*	0.788**	1	0.557**	0.212	-0.435*	0.431*	0.573**	0.754**	0.030	0.209	0.584**	0.709**	0.711**	0.542**
T	-0.121	0.440**	0.557**	1	0.200	-0.139	0.144	0.225	0.465**	-0.146	0.422*	0.331	0.463**	0.360*	0.160
AW	0.631**	0.339	0.212	0.200	1	-0.533**	0.537**	0.468**	0.219	-0.148	-0.393*	0.344*	0.386*	0.476**	0.634**
A21	-0.756**	-0.403*	-0.435*	-0.139	-0.533**	1	-0.999**	-0.236	-0.219	0.283	0.421*	-0.348*	-0.476**	-0.482**	-0.395*
A22	0.753**	0.398*	0.431*	0.144	0.537**	-0.999**	1	0.236	0.219	-0.299	-0.417*	0.344*	0.472**	0.476**	0.399*
TPC	0.443**	0.588**	0.573**	0.225	0.468**	-0.236	0.236	1	0.558**	-0.183	-0.087	0.637**	0.573**	0.637**	0.710**
TFC	0.271	0.836**	0.754**	0.465**	0.219	-0.219	0.219	0.558***	1	0.014	0.256	0.694**	0.650**	0.714**	0.642**
ARS	-0.180	0.049	0.030	-0.146	-0.148	0.283	-0.299	-0.183	0.014	1	0.393*	-0.040	-0.063	-0.091	-0.081
PH	-0.545**	0.161	0.209	0.422*	-0.393*	0.421**	-0.471*	-0.087	0.256	0.393*	1	-0.027	-0.067	-0.127	-0.163
DPPH	0.523**	0.708**	0.584**	0.331	0.344*	-0.348*	0.344*	0.637**	0.694**	-0.040	-0.027	1	0.800**	0.850**	0.660**
ABTS	0.523**	0.696**	0.709**	0.463**	0.386*	-0.476**	0.472**	0.573**	0.650**	-0.063	-0.067	0.800**	1	0.890**	0.673**
OH	0.624**	0.775**	0.711**	0.360*	0.476**	-0.482**	0.476**	0.637**	0.714**	-0.091	-0.127	0.850**	0.890**	1	0.755**
R	0.607**	0.723**	0.542**	0.160	0.634**	-0.395*	0.399*	0.710**	0.642**	-0.081	-0.163	0.660**	0.673**	0.755**	1

A, amylase activity, P, protease activity, R, reducing power, “*”, indicates the correlation is significant at 0.05, “**”, indicates the correlation is significant at 0.01.

Table S7. Correlation under the influence of fermentation time at constant moisture content.

Indicators	SDF	A	P	T	AW	A21	A22	TPC	TFC	ARS	pH	DPPH	ABTS	OH	R
35%-SDF	1	0.177	-0.099	0.254	0.447	0.287	0272	0.657	-0.158	0.253	0.457	0.017	0.205	0.157	0.157
45%-SDF	1	0.569	0.798*	0.164	-0.627	-0.085	-0.746	0.895**	0.603	0.680	0.009	0.478	0.430	0.690	0.809*
55%-SDF	1	0.503	0.119	-0.308	0.824*	0.114	-0.294	0.780*	0.470	0.662	-0.381	0.705	0.721	0.792*	0.792*
65%-SDF	1	0.119	0.198	-0.294	0.937**	-0.668	0.658	0.205	0.515	-0.515	-0.547	0.756*	0.306	0.697	0.697
75%-SDF	1	0.198	0.305	-0.274	0.955**	-0.404	0.867*	0.738	0.390	0.119	0.059	0.371	0.389	0.624	0.624
35%-A	0.177	1	0.902**	0.041	-0.684	-0.728	0.734	0.007	0.810*	-0.764*	0.446	0.504	0.744	0.936**	0.202
45%-A	0.569	1	0.842*	0.318	-0.628	-0.705	-0.622	0.703	0.902**	0.732	0.543	0.864*	0.776*	0.881**	0.870*
55%-A	0.503	1	0.852*	0.458	0.328	-0.285	0.161	0.809*	0.926**	0.693	0.336	0.884**	0.732	0.785*	0.722
65%-A	0.119	1	0.853*	0.670	0.280	-0.585	0.654	0.852*	0.886**	-0.735	0.515	0.662	0.638	0.781*	0.712
75%-A	0.198	1	0.828*	0.536	0.142	0.468	0.411	0.559	0.907**	-0.398	0.270	0.766*	0.723	0.825*	0.803*
35%-P	-0.099	0.902**	1	-0.063	0.876**	-0.757*	0.760*	-0.339	0.899**	-0.663	0.493	0.429	0.833*	0.775*	0.009
45%-P	0.798*	0.842*	1	0.452	-0.760*	-0.326	-0.837*	0.917**	0.895**	0.918**	0.561	0.644	0.755*	0.957**	0.877**
55%-P	0.437	0.852*	1	0.528	0.472	-0.541	0.453	0.791*	0.897**	0.492	0.109	0.760*	0.743	0.788*	0.663
65%-P	-0.022	0.853*	1	0.837*	0.043	-0.446	0.457	0.882**	0.738	-0.462	0.684	0.513	0.692	0.561	0.596
75%-P	0.305	0.828*	1	0.633	0.277	0.476	0.444	0.371	0.950**	0.011	0.564	0.959**	0.795*	0.857*	0.726

35%-T	0.254	0.041	-0.063	1	0.392	0.580	-0.567	0.575	0.321	-0.084	0.1	0.411	-0.168	0.002	0.097
45%-T	0.164	0.318	0.452	1	0.141	0.093	-0.193	0.156	0.416	0.676	0.212	0.316	0.580	0.608	0.193
55%-T	-0.308	0.458	0.528	1	-0.185	-0.289	0.325	0.150	0.369	-0.136	0.630	0.4	0.416	0.320	0.014
65%-T	-0.294	0.670	0.837*	1	-0.210	-0.326	0.325	0.696	0.542	-0.459	0.710	0.350	0.611	0.303	0.421
75%-T	-0.274	0.536	0.633	1	-0.138	0.452	0.132	0.134	0.644	-0.274	0.330	0.527	0.667	0.484	0.389
35%-A_W	0.447	-0.684	-0.876**	0.392	1	0.846*	-0.832*	0.684	-0.733	0.605	-0.323	-0.259	-0.702	-0.583	0.052
45%-A_W	-0.627	-0.628	-0.760*	0.141	1	0.470	0872*	-0.880**	-0.568*	-0.724	-0.572	-0.634	-0.468	-0.614	-0.660
55%-A_W	0.824*	0.328	0.472	-0.185	1	-0.311	0.137	0.799**	0.308	0.198	-0.696	0.488	0.640	0.766**	0.844**
65%-A_W	0.937**	0.280	0.043	-0.210	1	-0.769*	0.774*	0.191	0.611	-0.654	-0.594	0.734	0.363	0.811*	0.757*
75%-A_W	0.955**	0.142	0.277	-0.138	1	-0.497	0.888**	0.762*	0.393	0.171	0.117	-0.259	0.481	0.606	0.599
35%-A_{21}	0.287	-0.728	-0.757*	0.580	0.846*	1	-0.998**	0.429	-0.484	0.673	-0.076	-0.170	-0.566	-0.726	0.386
45%-A_{21}	-0.085	-0.705	-0.326	0.093	0.470	1	0.362	-0.310	-0.628	-0.231	-0.406	-0.825*	-0.534	-0.415	-0.385
55%-A_{21}	0.114	-0.285	-0.541	-0.289	-0.311	1	-0.960**	-0.418	-0.352	0.237	0.334	0.019	-0.015	-0.189	-0.255
65%-A_{21}	-0.668	-0.585	-0.446	-0.326	-0.769*	1	-0.990**	-0.582	-0.860*	0.833*	0.198	-0.803*	-0.390	-0.858*	-0.975**
75%-A_{21}	-0.404	0.468	0.476	0.452	-0.497	1	-0.182	-0.212	0.385	-0.119	0.546	0.347	-0.038	0.144	0.256
35%-A_{22}	-0.272	0.734	0.760*	-0.567	-0.832*	-0.998**	1	-0.391	0.486	-0.658	0.087	0.205	0.579	0.746	-0.408
45%-A_{22}	-0.746	-0.622	-0.837*	-0.193	0.872*	0.362	1	-0.895**	-0.721	-0.821*	-0.468	-0.524	-0.490	-0.718	-0.598

55%-A_{22}	-0.294	0.161	0.453	0.325	0.137	-0.960**	1	0.209	0.298	-0.341	-0.252	-0.174	-0.097	0.026	0.093
65%-A_{22}	0.658	0.654	0.457	0.325	0.774*	-0.990**	1	0.617	0.900**	-0.885**	-0.154	0.827*	0.410	0.899**	0.976**
75%-A_{22}	0.867*	0.411	0.444	0.132	0.888**	-0.182	1	0.916**	0.609	-0.191	0.114	0.429	0.572	0.793*	0.848*
35%-TPC	0.657	0.007	-0.339	0.575	0.684	0.429	-0.391	1	-0.194	0.126	0.012	0.369	-0.212	0.182	-0.332
45%-TPC	0.895**	0.703	0.917**	0.156	--0.880**	-0.310	-0.895**	1	0.817*	0.762*	0.413	0.583	0.630	0.813*	0.838*
55%-TPC	0.780*	0.809*	0.791*	0.150	0.799**	-0.418	0.209	1	0.719	0.528	-0.237	0.814*	0.772*	0.915**	0.927**
65%-TPC	0.205	0.852*	0.882**	0.696	0.191	-0.582	0.617	1	0.857*	-0.612	0.641	0.719	0.566	0.672	0.690
75%-TPC	0.738	0.559	0.371	0.134	0.762*	-0.212	0.916**	1	0.611	-0.472	-0.066	0.341	0.603	0.777*	0.865*
35%-TFC	-0.158	0.810*	0.899**	0.321	-0.733	-0.484	0.486	-0.194	1	0.675	0.480	0.618	0.684	0.693	-0.003
45%-TFC	0.603	0.902**	0.895**	0.387	-0.724	-0.628	-0.721	0.817*	1	0.180*	0.624	0.858*	0.935**	0.946**	0.815*
55%-TFC	0.470	0.926**	0.897**	0.369	0.308	-0.352	0.298	0.719	1	0.716	0.253	0.760*	0.666	0.697	0.678
65%-TFC	0.515	0.886**	0.738	0.542	0.611	-0.860*	0.900**	0.857*	1	-0.891**	0.226	0.887**	0.651	0.945**	0.935**
75%-TFC	0.390	0.907**	0.950**	0.644	0.393	0.385	0.609	0.611	1	-0.170	0.468	0.879**	0.864*	0.941**	0.878**
35%-ARS	0.253	-0.764*	-0.663	0.084	0.605	0.673	-0.659	0.126	-0.675	1	0.147	-0.278	-0.214	-0.702	0.440
45%-ARS	0.680	0.732	0.918**	0.532*	-0.572	-0.231	-0.821*	0.762*	0.780*	1	0.485	0.577	0.674	0.907**	0.661
55%-ARS	0.662	0.693	0.492	-0.136	0.198	0.237	-0.341	0.528	0.716	1	0.246	0.699	0.489	0.501	0.515
65%-ARS	-0.515	-0.735	-0.462	-0.459	-0.654	0.833*	-0.885**	-0.612	-0.891**	1	-0.020	-0.851*	-0.608	-0.855**	-0.858*

75%-ARS	0.119	-0.398	0.011	-0.274	0.171	-0.119	-0.191	-0.427	-0.170	1	0.613	-0.029	-0.155	-0.258	-0.314
35%-pH	0.457	0.446	0.493	0.1	-0.323	-0.076	0.087	0.012	0.480	0.147	1	0.491	0.848*	0.387	0.411
45%-pH	0.009	0.543	0.561	0.212	-0.634	-0.406	-0.468	0.413	0.624	0.485	1	0.286	0.545	0.543	0.363
55%-pH	-0.381	0.336	0.109	0.630	-0.696	0.334	-0.252	-0.237	0.253	0.246	1	0.272	0.088	-0.075	-0.313
65%-pH	-0.547	0.515	0.684	0.710	-0.594	0.198	-0.154	0.641	0.226	-0.020	1	0.043	0.308	-0.042	-0.066
75%-pH	0.059	0.270	0.564	0.330	0.117	0.546	0.114	-0.066	0.468	0.613	1	0.372	0.210	0.247	0.326
35%-DPPH	0.017	0.017	0.017	0.411	-0.259	-0.170	0.205	0.369	0.618	-0.278	0.491	1	0.490	0.672	-0.452
45%-DPPH	0.478	0.864*	0.664	0.316	-0.468	-0.825*	-0.524	0.583	0.858*	0.577	0.286	1	0.821*	0.767*	0.673
55%-DPPH	0.705	0.705	0.705	0.4	0.488	0.019	-0.174	0.814*	0.760*	0.699	0.272	1	0.921**	0.918**	0.767*
65%-DPPH	0.756*	0.756*	0.756*	0.350	0.734	-0.803*	0.827*	0.719	0.887**	-0.851*	0.043	1	0.692	0.895**	0.860*
75%-DPPH	0.371	0.371	0.371	0.527	0.299	0.347	0.428	0.341	0.879**	-0.029	0.372	1	0.781*	0.852*	0.641
35%-ABTS	0.205	0.744	0.833*	-0.168	-0.702	-0.566	0.579	-0.212	0.684	-0.214	0.848*	0.490	1	0.678	0.181
45%-ABTS	0.430	0.776*	0.755*	0.580	-0.475	-0.534	-0.490	0.630	0.935**	0.674	0.545	0.821*	1	0.891**	0.677
55%-ABTS	0.721	0.721	0.721	0.416	0.640	-0.015	-0.098	0.772*	0.666	0.489	0.087	0.921**	1	0.961**	0.806*
65%-ABTS	0.306	0.306	0.306	0.611	0.363	-0.390	0.410	0.566	0.651	-0.608	0.308	0.692	1	0.658	0.553
75%-ABTS	0.389	0.389	0.389	0.667	0.481	-0.038	0.572	0.603	0.864*	-0.155	0.210	0.781*	1	0.678	0.181
35%-OH	0.157	0.936*	0.775**	0.002	-0.583	-0.726	0.746	0.182	0.693	-0.702	0.387	0.672	0.678	1	-0.485

45%-OH	0.690	0.881**	0.957**	0.608	-0.614	-0.415	-0.718	0.813*	0.946**	0.907**	0.543	0.767*	0.891**	1	0.843
55%-OH	0.792*	0.785*	0.788*	0.320	0.766*	-0.189	0.026	0.915**	0.697	0.501	-0.075	0.918**	0.961**	1	0.909*
65%-OH	0.697	0.781*	0.561	0.303	0.811**	-0.857*	0.899**	0.672	0.945**	-0.855**	-0.042	0.895**	0.658	1	0.928**
75%-OH	0.624	0.825*	0.857*	0.484	0.606	0.144	0.793*	0.777*	0.941**	-0.702	0.247	0.852*	0.870*	1	0.916**
35%-R	0.275	-0.202	0.009	0.097	0.052	0.383	-0.408	0.332	-0.003	0.440	0.411	-0.452	0.181	-0.485	1
45%-R	0.809*	0.870**	0.877**	0.193	-0.660	-0.385	-0.598	0.838*	0.815*	0.661	0.363	0.673	0.677	0.843*	1
55%-R	0.867**	0.722	0.663	0.014	0.844*	-0.255	0.093	0.927**	0.678	0.515	-0.313	0.767*	0.806*	0.909**	1
65%-R	0.651	0.712	0.596	0.421	0.757	-0.957**	0.976**	0.690	0.935**	-0.858*	-0.066	0.860*	0.553	0.928**	1
75%-R	0.612	0.803*	0.726	0.389	0.599	0.256	0.848*	0.865*	0.878**	-0.314	0.326	0.641	0.686	0.916**	1

A, amylase activity; P, protease activity; R, reducing power; “*”, indicates the correlation is significant at 0.05; “**”, indicates the correlation is significant at 0.01.
